# MHC Class I Expression by Donor Hematopoietic Stem Cells Is Required to Prevent NK Cell Attack in Allogeneic, but Not Syngeneic Recipient Mice

**DOI:** 10.1371/journal.pone.0141785

**Published:** 2015-11-06

**Authors:** Yuichi Hirata, Hao-Wei Li, Kazuko Takahashi, Hiroshi Ishii, Megan Sykes, Joji Fujisaki

**Affiliations:** 1 Columbia Center for Translational Immunology, Department of Medicine, Surgery and Microbiology/Immunology, College of Physicians and Surgeons, Columbia University, New York, New York, United States of America; 2 Department of Pathology, Juntendo University School of Medicine, Tokyo, Japan; University of Kentucky, UNITED STATES

## Abstract

NK cells resist engraftment of syngeneic and allogeneic bone marrow (BM) cells lacking major histocompatibility (MHC) class I molecules, suggesting a critical role for donor MHC class I molecules in preventing NK cell attack against donor hematopoietic stem and progenitor cells (HSPCs), and their derivatives. However, using high-resolution *in vivo* imaging, we demonstrated here that syngeneic MHC class I knockout (KO) donor HSPCs persist with the same survival frequencies as wild-type donor HSPCs. In contrast, syngeneic MHC class I KO differentiated hematopoietic cells and allogeneic MHC class I KO HSPCs were rejected in a manner that was significantly inhibited by NK cell depletion. *In vivo* time-lapse imaging demonstrated that mice receiving allogeneic MHC class I KO HSPCs showed a significant increase in NK cell motility and proliferation as well as frequencies of NK cell contact with and killing of HSPCs as compared to mice receiving wild-type HSPCs. The data indicate that donor MHC class I molecules are required to prevent NK cell-mediated rejection of syngeneic differentiated cells and allogeneic HSPCs, but not of syngeneic HSPCs.

## Introduction

NK cells vigorously resist engraftment of syngeneic and allogeneic MHC class I KO BM cells [1–3], indicating a critical role for donor MHC class I molecules in suppressing NK cell attack. However, engraftment resistance may not simply reflect rejection of MHC class I KO HSPCs, but rather of their derivatives. Therefore, it still remains unexplored whether prevention of NK cell attack against donor HSPCs requires donor MHC class I molecules.

Our recent study suggests that tolerance against donor stem cells is achieved in a different manner from that against differentiated cells [4]. High-resolution *in vivo* microscopy (IVM) enabled us to reveal persistence of allogeneic HSPCs in non-conditioned immune competent recipient mice for an unexpectedly prolonged time (over 60 days), while differentiated cells were immediately rejected [4]. The data suggest that, compared to mature cells, HSPCs may have a limited susceptibility to immune attack, similar to embryonic and germline stem cells residing in immune privileged sites of the testis and the placenta [5–7].

We herein sought to determine whether MHC class I molecules of donor HSPCs [8] prevent NK cell attack. Instead of engraftment assays, we utilized IVM [4,9,10] to track the fate of MHC class I KO HSPCs and differentiated cells, respectively, following intravenous injection into allogeneic or syngeneic non-conditioned immune competent recipient mice. Allogeneic MHC class I KO HSPCs and syngeneic MHC class I KO hematopoietic differentiated cells were both rejected in a manner that was significantly inhibited by NK cell depletion. However, in contrast to the previous studies showing engraftment resistance of syngeneic MHC class I KO BM cells [1,2], we demonstrated that syngeneic MHC class I KO donor HSPCs persist with the same survival frequencies as wild-type donor HSPCs. Our data indicate that donor MHC class I molecules are required to prevent NK cell attack against allogeneic HSPCs and syngeneic differentiated cells, but not syngeneic HSPCs. Furthermore, a time-lapse live imaging approach enabled us to obtain a novel window into the dynamic interaction of donor HSPCs with NK cells *in vivo* and in real-time.

## Materials and Methods

### Animals

C57BL/6J, Balb/cJ, B10.A mice (Jackson Laboratory, Bar Harbor, ME), B6 H2-Kb H2-Db Double Knockout (B6.129P2-H2-Kb^tm1^ H2-Db^tm1^ N12), BALB/c Rag2^-/-^mice, and BALB/c Rag2^-/-^γ_c_^-/-^mice (Taconic, Hudson, NY) were housed in a specific pathogen-free environment. The mice were anaesthetized with 2–3% isoflurane and burpenorphine. The mice were sacrificed by CO2 inhalation and cervical dislocation. Studies were conducted with approval from Institutional Review Boards and Animal Care and Use Committees at Columbia University.

### Antibodies

FITC conjugated Lineage mAbs (B220, Mac1, GR-1, CD2, CD3a, CD8a, CD4, CD19 and Ter119), APC-eFluor 780 conjugated ckit mAbs, and FITC conjugated Granzyme B mAbs were purchased from eBioscience. PE conjugated CD49b mAbs, APC/Cy7 conjugated TCR-βmAbs, BV510 conjugated CD3 mAbs, and APC conjugated NK1.1 mAbs were purchased from Biolegend. BV605 conjugated Sca-I mAbs were purchased from BD Pharmingen.

### Isolation and transplantation of HSPCs and differentiated cells

Bone marrow cells were isolated by crushing femurs, tibias, iliac bones, and vertebrae. After centrifugation for 5 minutes at 1200 rpm, the cells were resuspended in 40% Percoll, and overlaid on 80% Percoll in a 15 ml Falcon tube. Percoll gradient separation was performed by centrifugation for 20 min at 2500 rpm at room temperature. Cells were collected from the interphase, washed once, and stained with the antibodies listed above. cKit+Sca1+Lin- HSPCs, or Lin+ differentiated cells were sorted using an Influx (Becton Dickinson), stained with 10 μM DiD (Invitrogen) in PBS with 0.1% fetal bovine serum for 20 min at 37°C, and washed twice in PBS with 0.1% fetal bovine serum. DiD-labeled HSPCs or Lin+ cells (30,000, or 500,000 cells per recipient, respectively) were injected into the tail vein of recipient mice.

### Transplantation of HSPCs or differentiated cells into mice depleted of NK cells

We transplanted DiD-labeled B6 wild-type or MHC class I KO HSPCs (30,000 cells per mouse) into non-irradiated allogeneic B10.A mice receiving anti-NK1.1 antibody treatment (0.2 mg/mouse) on day -5, -1, and 5. We transplanted DiD-labeled B6 wild-type or MHC class I KO Lin+ cells (500,000 cells per mouse) into non-irradiated B6 mice receiving anti-NK1.1 antibody treatment (0.2 mg/mouse) on day -1, and 5.

### Adoptive transfer of NK cells for the IVM study

18 hours after intraperitoneal injection of polyinosinic-polycytidylic acid (poly I:C) (100 μg/mouse), NK cells were isolated from Balb/cJ mice using the NK cell isolation kit (Miltenyi, Bergisch Gladbach, Germany). Subsequently, NK cells were stained with 10μM DiI in PBS with 0.1% fetal bovine serum for 20 min at 37°C, washed twice in PBS with 0.1% fetal bovine serum, and were injected (0.5 x 10^6^/mouse) into the tail veins of the mice.

### IVM

The mice were anaesthetized with 2–3% isoflurane and burpenorphine. A small incision was made in the scalp to expose the underlying skull bone, and then the mouse was placed on a custom-designed heated stage. The skull bone was kept intact. The temperature was maintained at 37°C by a heated pad placed under the mouse body. Imaging was performed using a Leica SP8 MP microscope (Leica Microsystems). Second harmonic microscopy was used to visualize the bone and to identify the major anatomical landmarks such as the central vein and the coronal suture. Bone rich in type-1 collagen was imaged by second harmonic generation using 870 nm excitation and 430 nm detection. Using the crossing of the central vein and coronal sutures as landmarks, we imaged identical areas of the skull (2540 μm (x) × 2570 μm (y) x 150 μm (z)) encompassing most of the parasagittal bone marrow cavities. The maximum imaging depth reaches 100–150 μm, which corresponds to 1/2~2/3 of the skull BM [9]. DiD signal was excited with a 633 nm laser and detected at 640–680 nm. DiI signal was excited with a 561 nm laser and detected with a 580–620 nm. Images were processed and analyzed with Imaris (Bitplane, version 5.7).

### Flow cytometry following intracellular staining of gramzyme B

Cells obtained from bones were incubated for 4–5 hour with Monesin and Leukocyte Activation Cocktail (BD bioscience) at 37°C, fixed and permeabilized with Cytofix/Cytoperm kit (BD Pharmingen). Subsequently, cells were stained with FITC-conjugated granzyme B mAbs (eBioscience). Flow cytometry was performed using an LSRII (BD Biosciences) or FACSCANTO (BD Biosciences) cytometer followed by analysis using FlowJo software (Tree Star Inc.).

### Statistics

Statistical analyses were performed with GraphPad Prism software (version 6.0). Statistical significance was determined by using 1-way analysis of variance (ANOVA) with Bonferroni posttest analysis or 2-tailed t-test test. P values lower than 0.05 were considered to be significant. All data are presented as mean ± SD.

## Results

### Transplantation of MHC class I KO HSPCs or differentiated cells

We sought to determine whether MHC class I molecules highly expressed by donor HSPCs [8] (S1 Fig) prevent NK cell attack. Using IVM, we tracked the fate of MHC class I KO or wild-type HSPCs following the intravenous injection into non-irradiated immune competent allogeneic or syngeneic recipients. cKit+Sca1+Lin- (KSL) HSPCs were isolated from B6 wild-type or H2-Kb H2-Db Double Knockout mice, labeled by lipophilic membrane dye, DiD, and injected into the tail veins of non-irradiated BALB/c or B6 recipient mice (0.03 million cells/mouse). On day 1 and 12, using IVM [4,9,10], we obtained 3D images of the skull BMs of the recipient mice.

**Fig 1:**
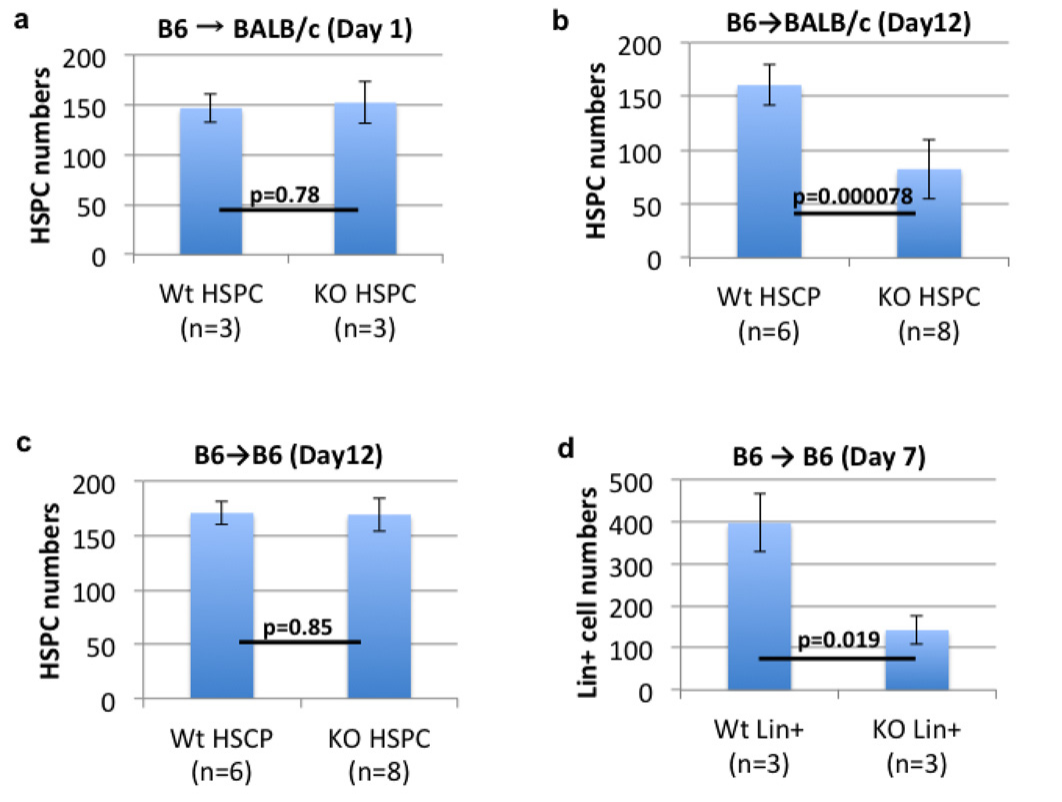
The lack of MHC class I expression by donor cells leads to rejection of allogeneic HSPCs and syngeneic differentiated cells, but not of syngeneic HSPCs. **(a, b)** The numbers of B6 MHC class I KO and wild-type HSPCs in the identical regions (2540 μm (x) × 2570 μm (y) x 150 μm (z)) of the skull BMs of non-irradiated BALB/c mice on day 1 (a: 3 recipients/group in an independent experiment), and day 12 (b: 8 vs 6 recipients/group pooled from three independent experiments). **(c)** The numbers of B6 MHC class I KO and wild-type HSPCs in the skull BMs of non-irradiated B6 mice on day 12 (8 vs 6 recipients/group pooled from three independent experiments). **(d)** The numbers of B6 MHC class I KO and wild-type lineage+ differentiated cells in the identical regions (2540 μm (x) × 2570 μm (y) x 150 μm (z)) of the skull BMs of B6 mice on day 7 (3 recipients/group in an independent experiment).

On day 1, the numbers of DiD-labeled B6 wild-type HSPCs and B6 MHC class I KO HSPCs in identical regions of the skull BMs were comparable in BALB/c recipient mice (Fig 1A). This indicates that the lack of MHC class I of donor HSPCs did not affect HSPC homing or rejection in the circulation.

Importantly, on day 12, the lack of MHC class I on donor HSPCs led to a 50% reduction in the numbers of donor HSPCs in allogeneic recipients (Fig 1B), but not a significant reduction in syngeneic recipients (Fig 1C, S2 Fig, S1 Table). Regardless of titration of the numbers of transplanted donor HSPCs, syngeneic MHC class I KO HSPCs survived with comparable frequencies with syngeneic wild-type HSPCs (S3 Fig). Additionally, the IVM study following intravenous injection of MHC class I KO differentiated (lineage+) cells into syngeneic B6 recipients showed a significant rejection of syngeneic MHC class I KO differentiated cells (Fig 1D). The data indicate that MHC class I expression by donor cells are required for preventing rejection of allogeneic HSPCs and syngeneic mature cells, but not of syngeneic HSPCs.

The survival frequencies of syngeneic MHC class I KO HSPCs were not significantly altered by the simultaneous injection of syngeneic MHC class I KO BM cells that will activate NK cells (S4 Fig). Therefore, the previously demonstrated engraftment resistance of syngeneic MHC class I KO BM cells [1,2] may be attributed to the rejection of MHC class I KO donor differentiated cells, but not of MHC class I KO stem cells.

### The role of NK cells in the rejection of syngeneic MHC class I KO differentiated cells and allogeneic MHC class I KO HSPCs

**Fig 2:**
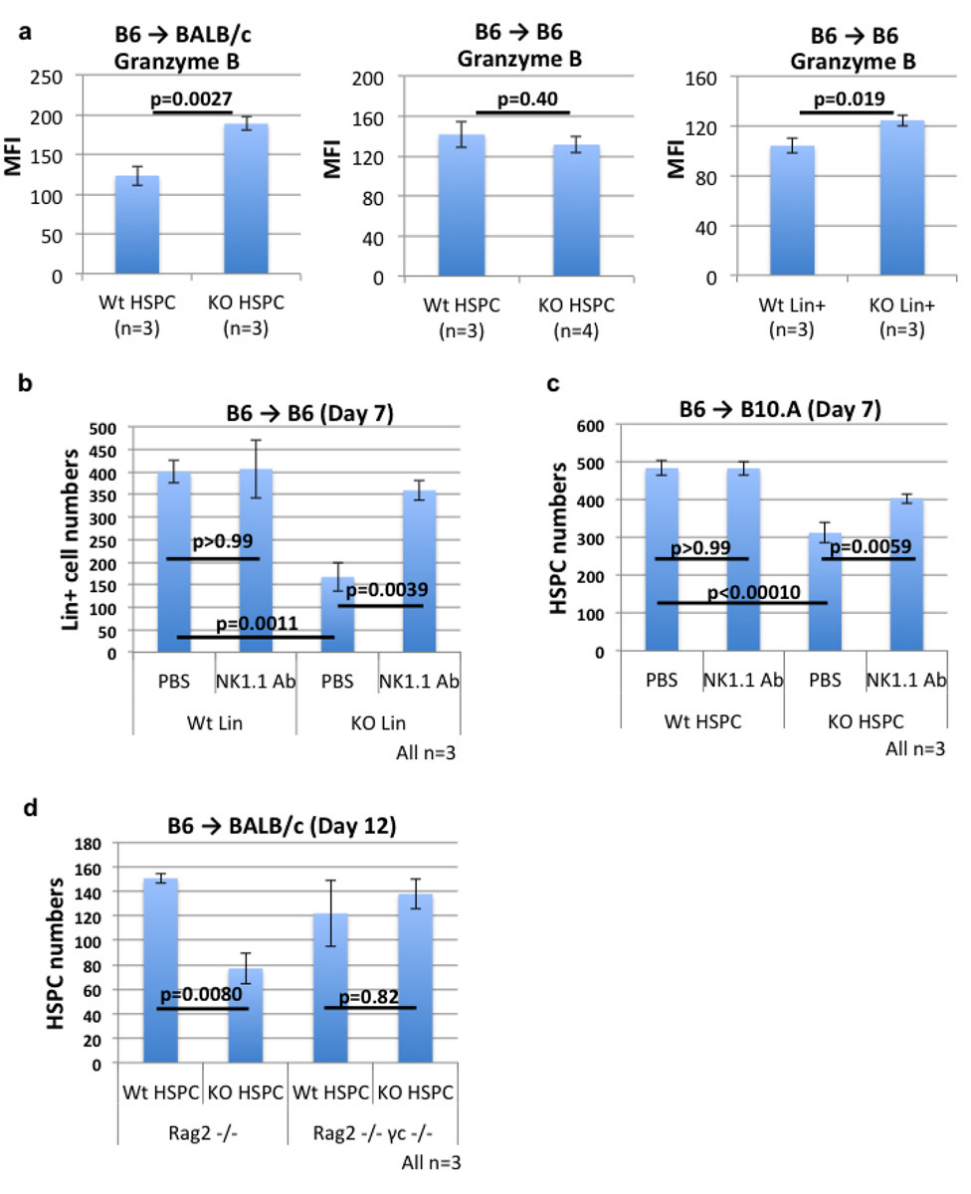
NK cells reject allogeneic MHC class I KO HSPCs and syngeneic MHC class I KO differentiated cells, but not syngeneic MHC class I KO HSPCs. **(a)** Flow cytometry analysis of granzyme-B expression in NK cells in BALB/c mice transplanted with B6 MHC class I KO or wild-type HSPCs (left), in B6 mice transplanted with B6 MHC class I KO or wild-type HSPCs (middle), and in B6 mice transplanted with B6 MHC class I KO or wild-type differentiated cells (right). MFI: mean fluorescence intensity. 3 recipients/group were analyzed in an independent experiment. **(b)** The numbers of B6 MHC class I KO and wild-type lineage+ cells in the identical regions (2540 μm (x) × 2570 μm (y) x 150 μm (z)) of the skull BMs of B6 mice receiving PBS or anti-NK1.1 antibody treatment. 3 recipients/group were analyzed in an independent experiment. **(c)** The numbers of MHC class I KO HSPCs persisting in non-irradiated B10.A mice treated with PBS or ani-NK1.1 antibody on day 7. 3 recipients/group were analyzed in an independent experiment. **(d)** The numbers of MHC class I KO HSPCs persisting in non-irradiated RAG2 -/- mice and RAG2 -/- ϒc -/- mice on day 12. 3 recipients/group were analyzed in an independent experiment.

We next elucidated the role of NK cells in rejecting syngeneic MHC class I KO differentiated cells and allogeneic MHC class I KO HSPCs. Flow cytometry analysis showed that granzyme-B expression by BM NK cells is significantly up-regulated by transplantation of syngeneic MHC class I KO differentiated cells and allogeneic MHC class I KO HSPCs, but not of syngeneic MHC class I KO HSPCs (Fig 2A). Anti-NK1.1 antibody treatment significantly reversed the decrease in the numbers of both syngeneic MHC class I KO differentiated cells (Fig 2B) and allogeneic MHC class I KO HSPCs (Fig 2C). This indicates that NK cells play a critical role in rejecting syngeneic MHC class I KO differentiated cells and allogeneic MHC class I KO HSPCs.

To further determine whether NK cells are sufficient for rejecting allogeneic MHC class I KO HSPCs, we compared the numbers of surviving B6 MHC class I KO HSPCs in non-irradiated BALB/c T-, B-, NK-cell deficient Rag2^-/-^γ_c_^-/-^mice and BALB/c T-, B-cell deficient Rag2^-/-^mice. The lack of MHC class I expression by donor HSPCs significantly reduced the numbers of donor cells surviving in Rag2-/- mice, but not in Rag2-/-γc-/- mice (Fig 2D). Taken together with the result of the study using anti-NK1.1 antibody treatment, the data indicate that MHC class I of donor HSPCs plays a critical role in suppressing NK cell-mediated rejection in allogeneic recipients.

### *In vivo* time-lapse imaging of NK cells and allo-HSPCs

**Fig 3:**
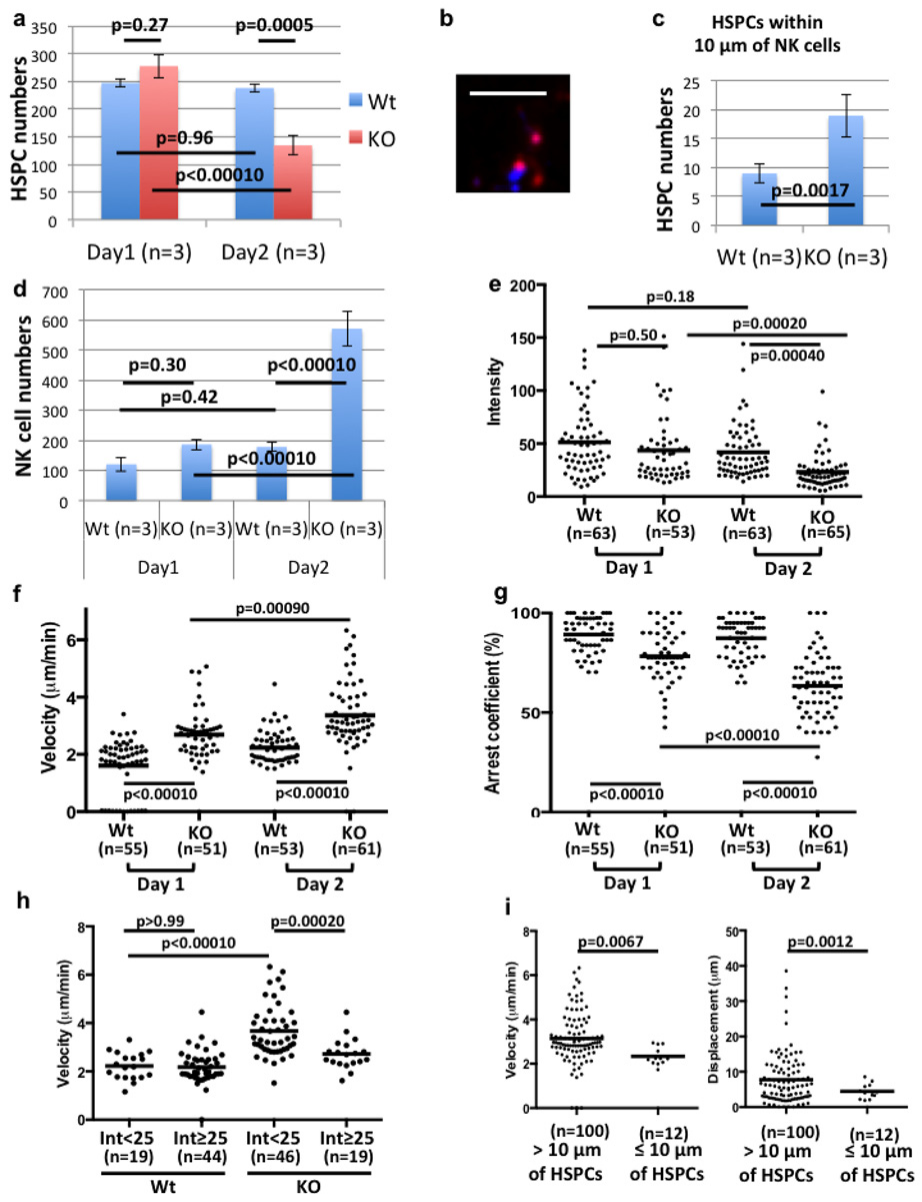
*In vivo* time-lapse imaging of NK cells and allo-HSPCs in the BM. **(a)** The numbers of B6 MHC class I KO or wild-type HSPCs in the skull BMs (2540 μm (x) × 2570 μm (y) x 150 μm (z)) of BALB/c mice 1 and 2 days after the HSPC transplantation. 3 recipients/group were analyzed in an independent experiment. **(b)** IVM showing direct contact of NK cells (blue) with MHC class I KO HSPCs (red). The scale bar indicates 100 μm. **(c)** The numbers of HSPCs within 10 μm of NK cells in the identical regions (2540 μm (x) x 2570 μm (y) x 150 μm (z)) of the skull BMs. 3 recipients/group were analyzed in an independent experiment. **(d)** The numbers of NK cells in the identical regions (800 μm (x) x 800 μm (y) x 150 μm (z)) of the skull BMs. **(e)** The intensity level (Relative fluorescence unites (RFU)) of DiI of NK cells. **(f)** The velocity of NK cells. **(g)** The arrest coefficient (the proportion of time in which NK cells do not move (velocity <2 μm)). **(h)** The correlation of the DiI signal levels with the velocity of NK cells. **(i)** The velocity and the displacement of NK cells located within or more than 10 μm of HSPCs. The data were pooled from the imaging of at least 3 recipients/group (d-i).

We next utilized a time-lapse *in vivo* imaging approach to determine how MHC class I of HSPCs influences the cellular dynamics of NK cells in live mice. NK cells were isolated from BALB/c mice following intraperitoneal injection of polyinosinic:polycytidylic acid (poly I:C), labeled by lipophilic membrane dye, DiI, and intravenously injected into non-irradiated BALB/c mice (0.5 million cells/mouse). 24 hours after the injection of BALB/c NK cells, non-irradiated BALB/c mice were intravenously injected with DiD-labeled B6 MHC class I KO or wild-type HSPCs (0.08 million cells/mouse). The skull BMs of live recipient mice were imaged by IVM 1 and 2 days after the injection of allogeneic HSPCs. The numbers of allogeneic MHC class I KO HSPCs on day 2 decreased up to 50% compared to day 1, while the numbers of wild-type HSPCs did not significantly change (Fig 3A). MHC class I KO HSPCs frequently form clusters with NK cells, as compared to wild-type HSPCs (Fig 3B and 3C).

The NK cell numbers in the skull BMs increased over time more robustly in mice with allogeneic MHC class I KO HSPCs compared to those with wild-type HSPCs (Fig 3D).

The signal levels of the membrane dye, DiI of NK cells significantly decreased over time in mice with MHC class I KO HSPCs, but not in mice with wild-type HSPCs (Fig 3E). Because these NK cells were imaged at the comparable imaging depth (within 20 μm of the endosteal bone surface) (S5 Fig), the low level of DiI signals of NK cells suggests the dye dilution following rapid proliferation of NK cells in mice with MHC class I KO HSPCs. The data indicate that NK cell proliferation was promoted in mice receiving allogeneic MHC class I KO HSPCs.

The time-lapse movie showed that HSPCs are stationary compared to NK cells in all mouse groups (S1 Video). MHC class I KO HSPCs more robustly activated the motility of NK cells than wild-type HSPCs, which is indicated by the increase in the velocity and the decrease of the arrest coefficient (the proportion of time in which NK cells do not move (velocity <2 μm/min)) (Fig 3F and 3G, S2 and S3 Videos). Moreover, the velocity of NK cells with low DiI signals was higher than that of NK cells with high DiI signals (Fig 3H, S5 Fig, S2 Video). This suggests that, following proliferation, NK cells became highly motile and acquired high immune surveillance activity. Taken together, the data suggest that mice receiving allogeneic MHC class I KO HSPCs showed a significant increase in NK cell motility and proliferation as well as frequencies of NK cell contact with and killing of HSPCs as compared to mice receiving wild-type HSPCs.

Although the previous imaging study revealed that NK cells in the tumor site repeat contact with target cells prior to killing [11], NK cells in the BM formed stable contact with MHC class I KO HSPCs and barely moved during the time-lapse movie recorded for more than 45 minutes (Fig 3I, S1 Video).

## Discussion

The previous studies demonstrated NK cell-mediated engraftment resistance of both syngeneic and allogeneic MHC class I KO BM cells [1–3]. But these studies did not rule out that the possibility that only MHC class I KO donor differentiated cells, but not HSPCs, are rejected. Our IVM study to track the fate of donor HSPCs and mature cells revealed persistence of syngeneic MHC class I KO HSPCs, while NK cells rejected syngeneic MHC class I KO differentiated cells and allogeneic MHC class I KO HSPCs. The data indicates that donor MHC class I molecules are not required to prevent NK cell attack against syngeneic HSPCs, but are against allogeneic HSPCs and syngeneic differentiated cells. The critical role for donor MHC class I molecules in suppressing NK cell attack against allogeneic HSPCs is supported by other studies showing that NK cells only weakly resist long-term engraftment of allogeneic BM cells [12], as well as engraftment following transplantation of allogeneic purified HSPCs [13].

NK cells may recognize the allogeneicity of MHC class I KO HSPCs through non-self recognition by monocytes [14,15] that secret inflammatory cytokines, which may directly activate NK cells [16] or induce up-regulation of NKG2D ligands by donor HSPCs [17]. Therefore, we cannot exclude the possibility that MHC class I molecules of allogeneic donor HSPCs indirectly suppress NK cells via monocytes or other cells. The failure of NK cells to reject syngeneic MHC class I KO HSPCs may be due to enrichment of FoxP3+ regulatory T cells around donor HSPCs that was demonstrated to allow allo-HSPC persistence in immune competent recipients [4]. Our study using IVM established a novel experimental model that allows the investigation of the dynamic interaction of immune cells with HSPCs in live mice. Future studies are warranted to investigate the role of MHC class I expression in NK cell self-tolerance against endogenous HSPCs.

## Supporting Information

S1 Fig MHC class I expression of cKit+Sca1+Lin- (KSL HSPCs) and spleen B220+ cells. (PNG)

S2 Fig IVM of B6 MHC class I KO HSPCs (right, red) and wild-type HSPCs (left, red) in the skull BMs of non-irradiated BALB/c (above) or B6 (below) mice on day 12. (PNG)

S3 Fig Syngeneic MHC class I KO HSPCs survived with comparable frequencies with syngeneic wild-type HSPCs, regardless of titration of the numbers of transplanted donor HSPCs. Following injection of 10,000 or 20,000 HSPCs per recipient, 3 recipients/group were analyzed in an independent experiment. Following injection of 30,000 HSPCs per recipient, 8 or 6 recipients/group pooled from three independent experiments were analyzed.
(PNG)

S4 Fig The numbers of DiD-labeled MHC class I KO HSPCs on day 12 were not significantly altered by the co-injection of MHC class I KO BM cells. DiD-labeled B6 MHC class I KO HPSCs (30,000/mouse) and non-labeled B6 MHC class I BM cells (5,000,000/mouse) were simultaneously injected into the tail veins of syngeneic B6 recipients. The data were pooled from at least two independent experiments.
(PNG)

S5 Fig The depth of imaged NK cells for the study of dye dilution. **(a) The depth of NK cells from the endosteal bone surface (**μ**m). (b) The depth of NK cells with high DiI signals (RFU≥25) or low DiI signals (RFU<25).** Red bar: average. The data were pooled from the imaging of at least 3 recipients/group.
(PNG)

S1 Table The numbers of B6 MHC class I KO and wild-type HSPCs in the identical regions (2540 μm (x) × 2570  m (y) x 150  m (z)) of the skull BMs of BALB/c mice (above), and of B6 mice on day 12 (below) on day 12. Three independent experiments per group were performed.
(PNG)

S1 Video Time-lapse movie of NK cells (green) interacting with allogeneic MHC class I KO HSPCs (red). (MOV)

S2 Video Time-lapse movie of BM NK cells (green) in mice receiving allogeneic MHC class I KO HSPCs. NK cells with high DiI signals (RFU≥25) are tracked by the red line, and NK cells with low DiI signals (RFU<25) are tracked by the blue line.
(MOV)

S3 Video Time-lapse movie of BM NK cells (green) in mice receiving allogeneic wild-type HSPCs. (MOV)
