# Supplementary figures and images for: MHC Class I Expression by Donor Hematopoietic Stem Cells Is Required to Prevent NK Cell Attack in Allogeneic, but Not Syngeneic Recipient Mice
Source: PLoS One. 2015 Nov 6;10(11):e0141785. doi: 10.1371/journal.pone.0141785 (PMC4636372; doi:10.1371/journal.pone.0141785)

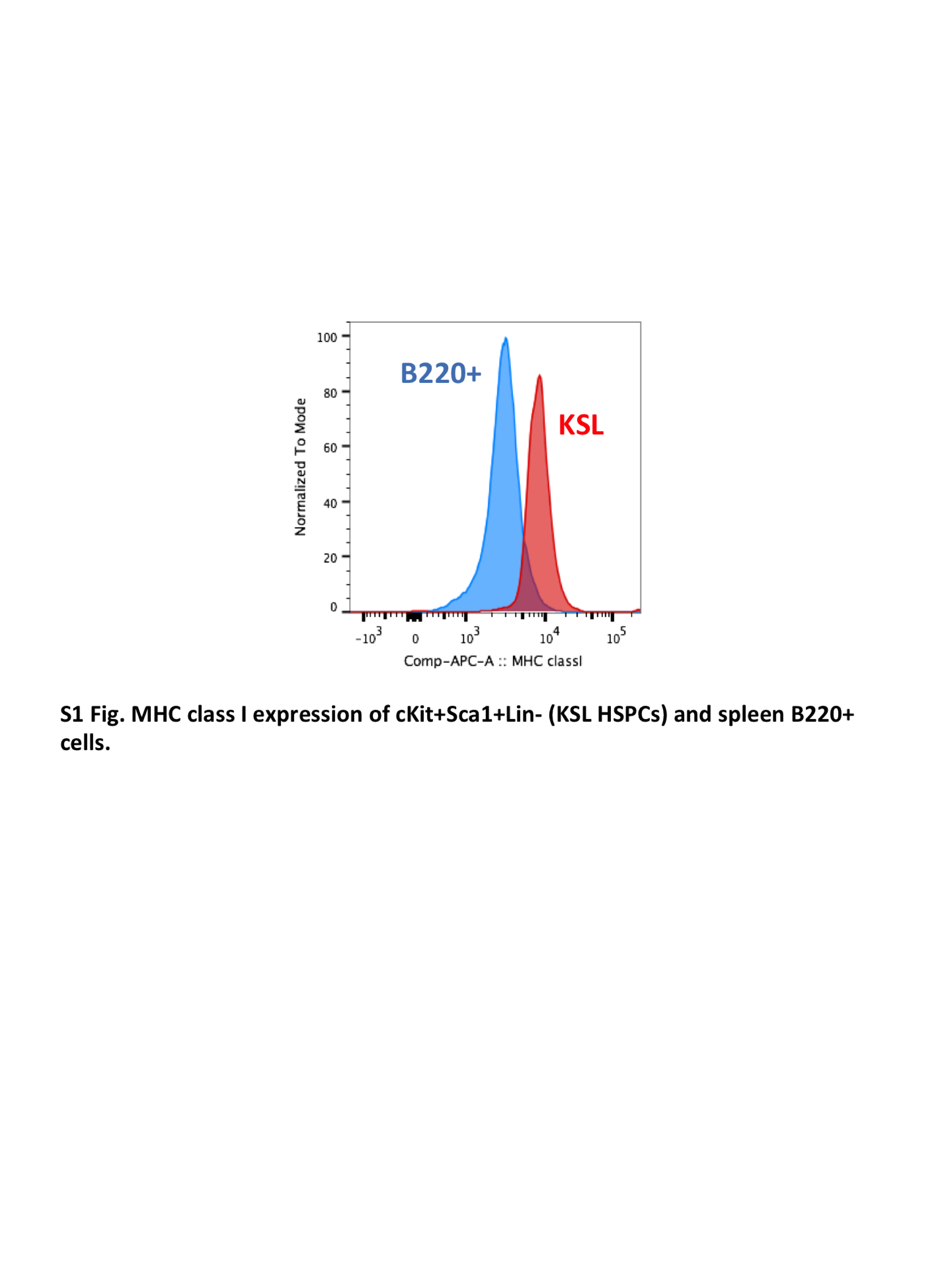

Supplement: S1 Fig — (PNG) [file pone.0141785.s001.png]

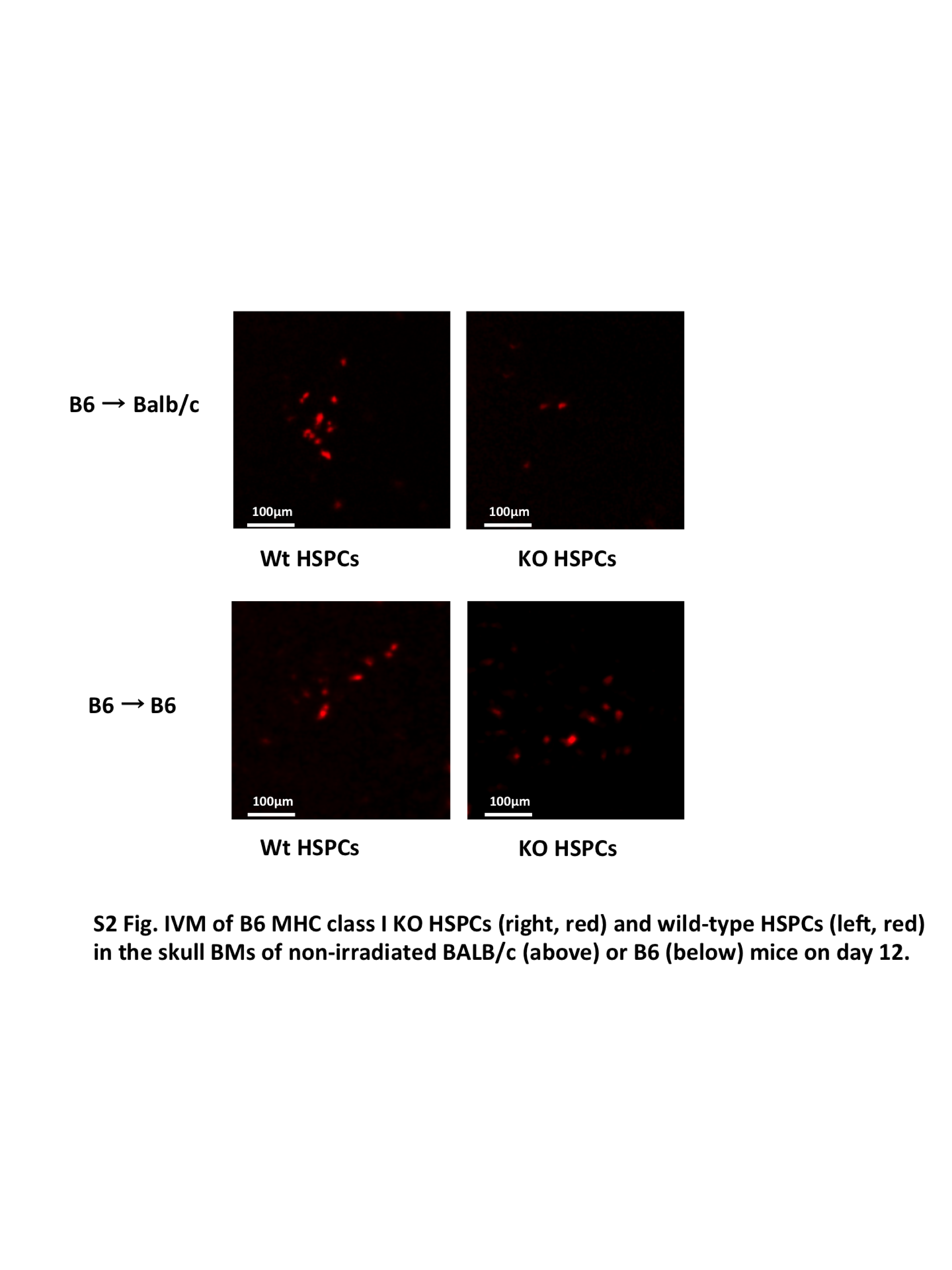

Supplement: S2 Fig — (PNG) [file pone.0141785.s002.png]

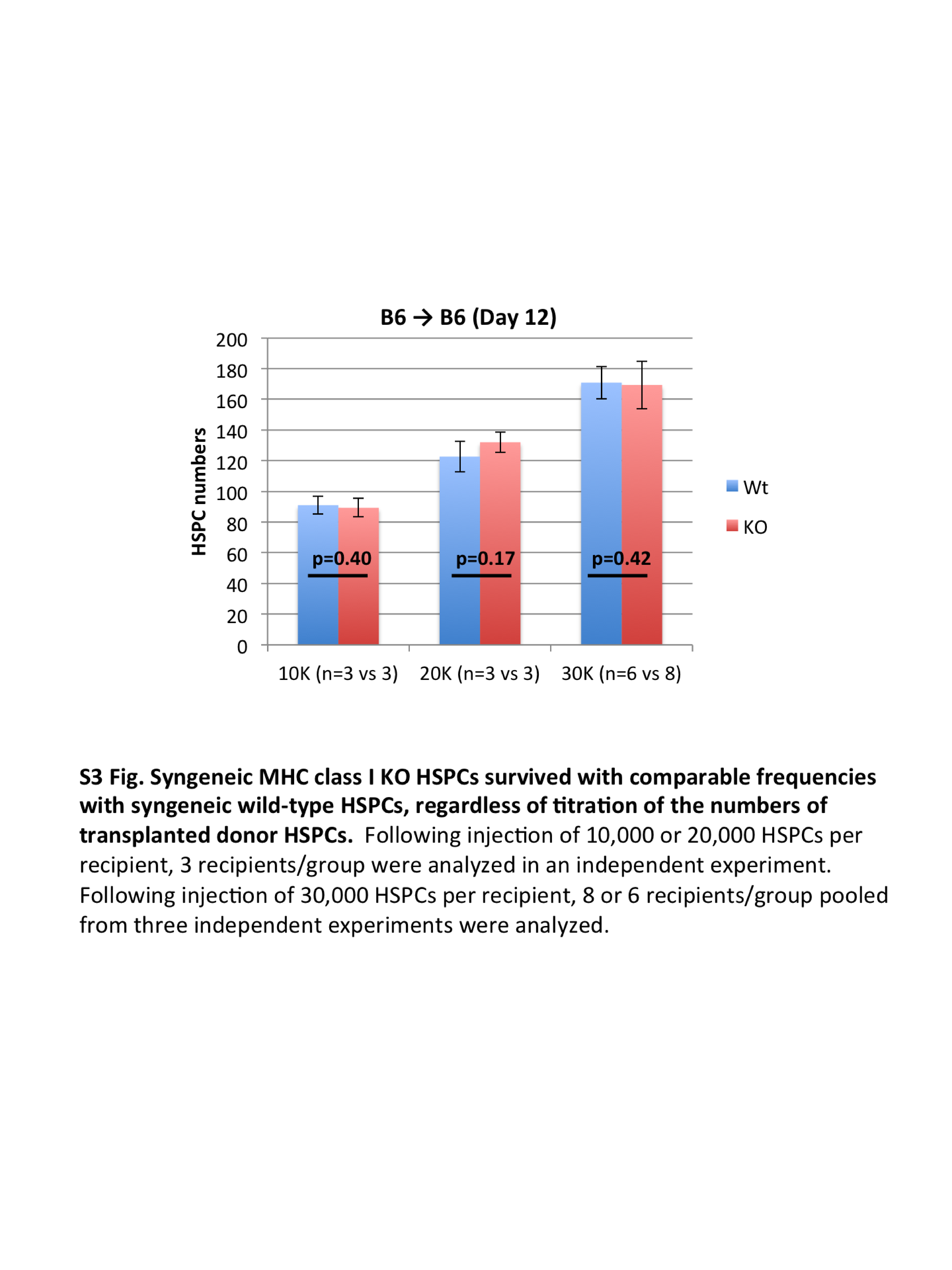

Supplement: S3 Fig — Following injection of 10,000 or 20,000 HSPCs per recipient, 3 recipients/group were analyzed in an independent experiment. Following injection of 30,000 HSPCs per recipient, 8 or 6 recipients/group pooled from three independent experiments were analyzed. (PNG) [file pone.0141785.s003.png]

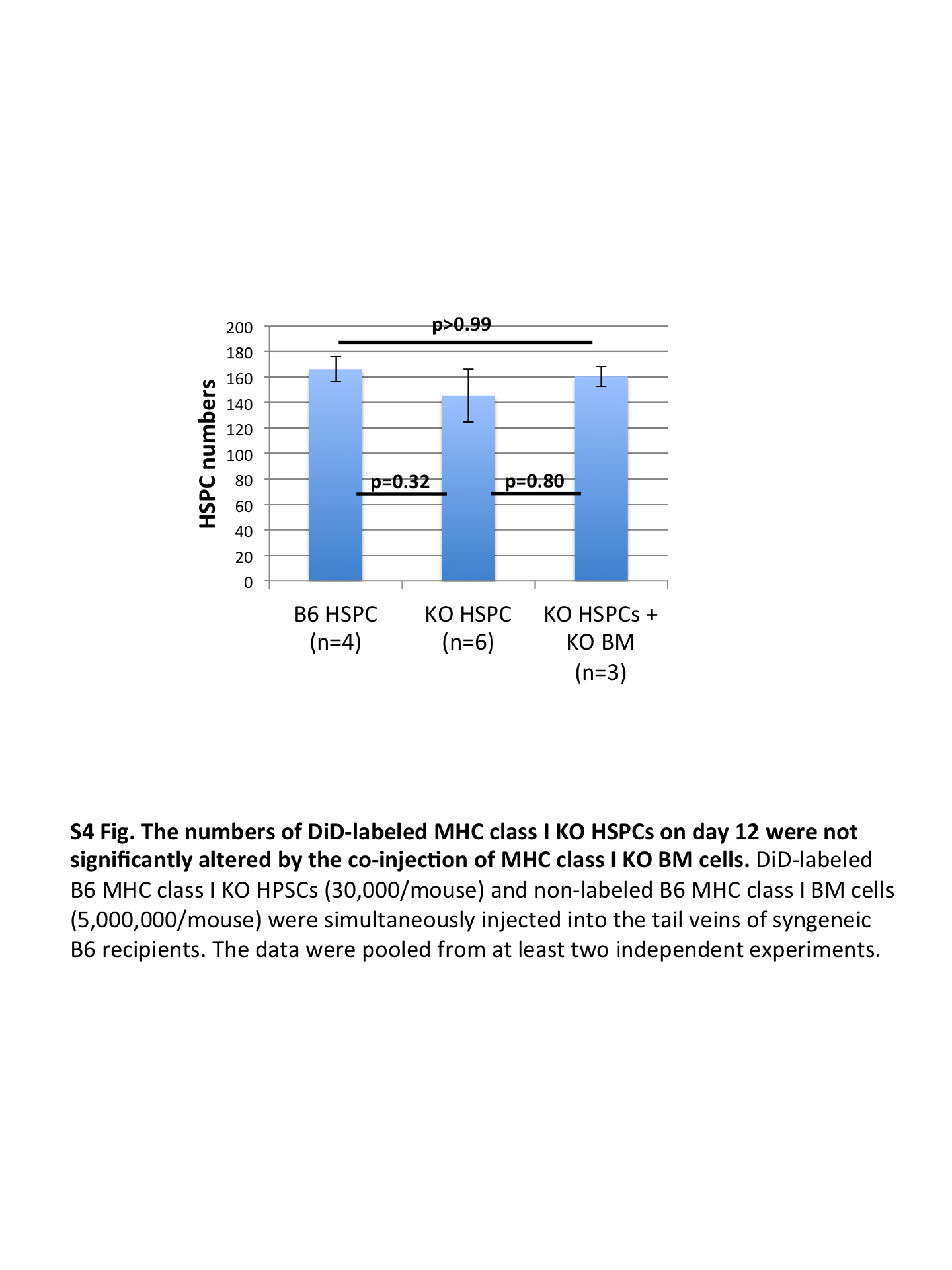

Supplement: S4 Fig — DiD-labeled B6 MHC class I KO HPSCs (30,000/mouse) and non-labeled B6 MHC class I BM cells (5,000,000/mouse) were simultaneously injected into the tail veins of syngeneic B6 recipients. The data were pooled from at least two independent experiments. (PNG) [file pone.0141785.s004.png]

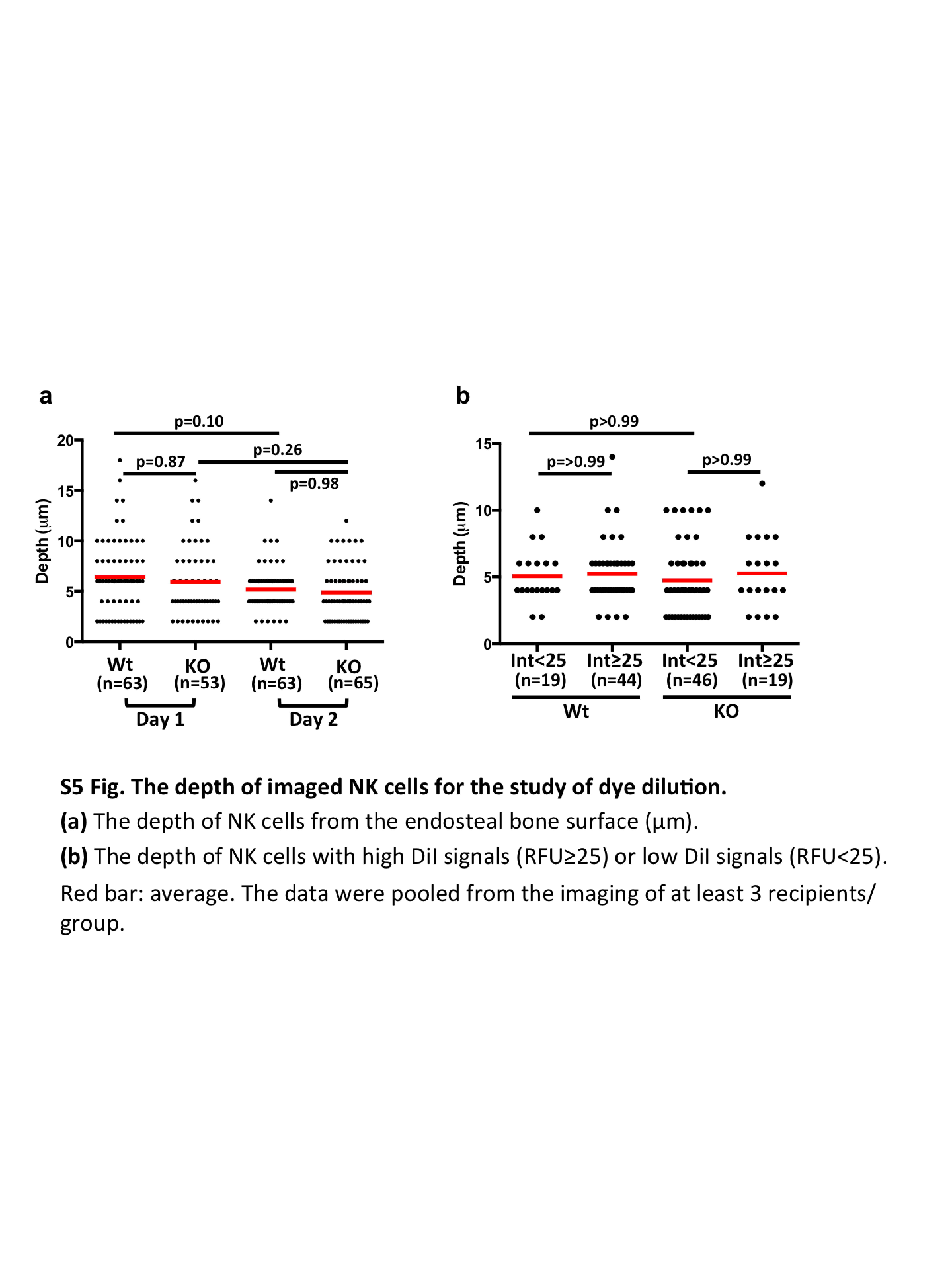

Supplement: S5 Fig — (a) The depth of NK cells from the endosteal bone surface (μm). (b) The depth of NK cells with high DiI signals (RFU≥25) or low DiI signals (RFU<25). Red bar: average. The data were pooled from the imaging of at least 3 recipients/group. (PNG) [file pone.0141785.s005.png]

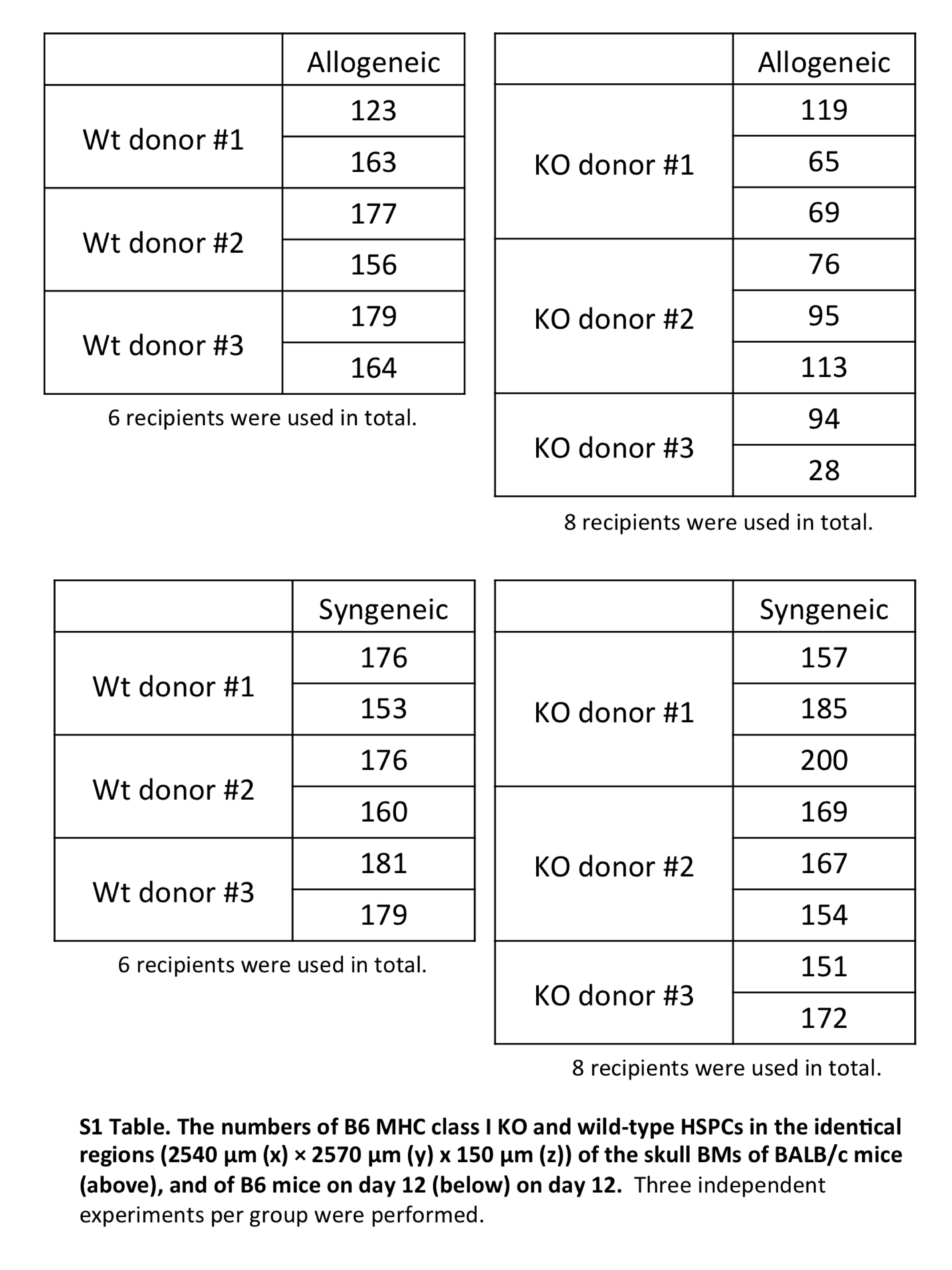

Supplement: S1 Table — Three independent experiments per group were performed. (PNG) [file pone.0141785.s006.png]
